# EHMTI-0125. Studying the permeability of the blood-brain barrier during migraine attacks using [11C]-dihydroergotamine

**DOI:** 10.1186/1129-2377-15-S1-F22

**Published:** 2014-09-18

**Authors:** C Schankin, F Maniyar, Y Seo, S Kori, M Eller, J Blecha, S Murphy, T Sprenger, H VanBrocklin, P Goadsby

**Affiliations:** 1 Department of Neurology, University of Munich Hospital - Großhadern, Munich, Germany; 2 Department of Neurology, The Royal Hospital London, London, UK; 3 Department of Radiology and Biomedical Imaging, University of California San Francisco, San Francisco, USA; 4 Allergan Inc., MAP Pharmaceuticals Inc., Mountain View, USA; 5 Department of Neurology, University of California San Francisco, San Francisco, USA; 6 Department of Neurology and Division of Neuroradiology, University Hospital Basel, Basel, Switzerland; 7 Headache Group, NIHR-Wellcome Trust Clinical Research Facility King's College London, London, UK

## Introduction

Due to unfavorable molecular size and lipophilicity, migraine-specific medications such as dihydroergotamine (DHE) are not expected to penetrate the blood-brain barrier (BBB). A breakdown of the BBB during migraine attacks has been postulated as the mechanism in which DHE accesses postulated central sites of action.

## Aim

To demonstrate whether the permeability of the BBB increases for DHE during migraine attacks.

## Methods

As a measure of parenchymal binding in the brain and thus BBB penetration, we calculated the influx rate constant Ki for the radioligand [11C]-dihydroergotamine ([11C]-DHE) using arterial blood input function over the course of dynamic positron emission tomography (PET). The influence of migraine on the Ki maps, i.e. the BBB was assessed in a second [11C]-DHE scan during glyceryl trinitrate (GTN)-induced migraine attacks.

## Results

Independent from the presence of migraine headache, six migraineurs and six age- and gender-matched control subjects showed identical binding of [11C]-DHE at the choroid plexus, the pituitary gland, and the venous sinuses. There was no binding (Ki = 0/min) in the brain parenchyma, including the candidate brainstem sites of action during migraine (periaqueductal grey, raphe nuclei) and the area with the highest density of the highest-affinity DHE receptors (hippocampus).

## Conclusions

The lack of ictal binding of [11C]-DHE to the brain parenchyma suggests that the BBB remains intact for DHE during migraine attacks. The efficacy of DHE in treating an acute migraine attack may have a peripheral component although some implicated structures remain outside the BBB.

